# Stem Cell Therapy and Curcumin Synergistically Enhance Recovery from Spinal Cord Injury

**DOI:** 10.1371/journal.pone.0088916

**Published:** 2014-02-18

**Authors:** D. Ryan Ormond, Craig Shannon, Julius Oppenheim, Richard Zeman, Kaushik Das, Raj Murali, Meena Jhanwar-Uniyal

**Affiliations:** 1 Department of Neurosurgery, New York Medical College, Valhalla, New York, United States of America; 2 Department of Cell Biology and Anatomy, New York Medical College, Valhalla, New York, United States of America; University of South Florida, United States of America

## Abstract

Acute traumatic spinal cord injury (SCI) is marked by the enhanced production of local cytokines and pro-inflammatory substances that induce gliosis and prevent reinnervation. The transplantation of stem cells is a promising treatment strategy for SCI. In order to facilitate functional recovery, we employed stem cell therapy alone or in combination with curcumin, a naturally-occurring anti-inflammatory component of turmeric (*Curcuma longa*), which potently inhibits NF-κB. Spinal cord contusion following laminectomy (T9–10) was performed using a weight drop apparatus (10 g over a 12.5 or 25 mm distance, representing moderate or severe SCI, respectively) in Sprague-Dawley rats. Neural stem cells (NSC) were isolated from subventricular zone (SVZ) and transplanted at the site of injury with or without curcumin treatment. Functional recovery was assessed by BBB score and body weight gain measured up to 6 weeks following SCI. At the conclusion of the study, the mass of soleus muscle was correlated with BBB score and body weight. Stem cell therapy improved recovery from moderate SCI, however, it had a limited effect on recovery after severe SCI. Curcumin stimulated NSC proliferation *in vitro*, and in combination with stem cell therapy, induced profound recovery from severe SCI as evidenced by improved functional locomotor recovery, increased body weight, and soleus muscle mass. These findings demonstrate that curcumin in conjunction with stem cell therapy synergistically improves recovery from severe SCI. Furthermore, our results indicate that the effect of curcumin extends beyond its known anti-inflammatory properties to the regulation of stem cell proliferation.

## Introduction

Acute traumatic spinal cord injury (SCI) occurs at an estimated annual incidence of 15–40 cases per million [1]. The prevalence of SCI is estimated at 1.275 million people (0.4% of the population) in the United States alone [2]. Much of the morbidity associated with SCI occurs due to the limited intrinsic ability of the spinal cord to recover following transection or contusion. The pathophysiology of SCI is considered biphasic in nature. Primary injury results from mechanical force injuring the spinal cord, tearing axons, blood vessels, and causing cell membrane disruption. Secondary injury occurs via the subsequent edema, ischemia, inflammation, cytokine production, free radical damage, glial scar formation, apoptosis and necrosis that ensue. Perilesional astrocytes alter their phenotype and over the weeks following SCI form the glial scar composed of astrocytes and a dense extracellular matrix [3]. Therapeutic modalities that promote recovery could be better understood by elucidating the pathophysiology of SCI. Necrosis, apoptosis, and inflammation play critical roles in defining neuronal cell death and formation of the glial scar implicated in limiting SCI recovery. In addition, secreted inflammatory cytokines and growth factors generally up-regulate pro-survival molecules, such as NF-κB [4]. Thus, the major hurdles in complete recovery from SCI are mostly due to the upregulation of certain inflammatory molecules after injury that result in gliosis. Curcumin (diferuloylmethane), the active ingredient in turmeric (*Curcuma longa*), is a highly pleiotropic molecule with potent anti-inflammatory properties. The pleiotropic activities attributed to curcumin because of its chemical structure appear to influence multiple signaling pathways activated in SCI [5], [6], [7]. Some of these signaling pathways include phospholipase, lipooxygenase, cyclooxygenase 2, leukotrienes, thromboxane, prostaglandins, nitric oxide, collagenase, elastase, hyaluronidase, monocyte chemoattractant protein-1 (MCP-1), interferon-inducible protein, tumor necrosis factor (TNF), interleukin-12 (IL-12), and NF-κB [6], [7], [8]. It has also been shown to reduce the inflammatory cascade and may help prevent plaque deposition and protein oxidation in neurodegenerative conditions [6]. Curcumin’s benefits in SCI have been recently reported in the literature, and this benefit may be due to a reduction in secondary injury to the spinal cord [9]–[16]. Furthermore, a study using curcumin with other dietary compounds showed enhancement of sensorimotor function, especially after exercise [15]. This suggests that curcumin added to the diet has a beneficial effect on recovery. In addition, the molecular mechanism underlying curcumin as a dietary supplement with Omega-3 fatty acids (DHA) has suggested that secondary injury can be prevented with such a treatment [16].

Stem cell therapies offer a promising way to replace neurons and oligodendrocytes lost to necrosis or apoptosis, help remyelinate axons, and increase the availability of neurotrophic factors to aid in local recovery [17]–[26]. Furthermore, stem cell therapy provides support to the surrounding milieu after injury to enhance axonal regeneration and/or neuroprotection. Transplantation of *in vitro* expanded NSC into rat spinal cord after contusion injury induced their differentiation into neurons or oligodendrocytes, and promoted functional recovery of limb movement [23], [26]. Also, restoration of neurotrophic factors seen in stem cells may aid in supporting the surrounding milieu after injury in order to enhance axonal regeneration and neuroprotection [27]. Stem cell therapy has been explored in animal models as an aid to recovery following SCI. Curcumin, a potent anti-inflammatory compound, has been shown to improve functional recovery from SCI [5]–[14]. However the use of curcumin with stem cell therapy has not been well defined. The purpose of this study is to investigate stem cell therapy with or without curcumin in treatment of moderate as well as severe SCI. Herein we provide evidence that the combined use of stem cell therapy with curcumin aid in recovery after thoracic SCI in an animal model.

## Materials and Methods

### Animals

Animal work was performed with the approval of the New York Medical College IACUC under approval number: 137-1-1210.1. Adult female Sprague-Dawley rats (200–250 grams; Charles River, Troy, NY) were maintained on a routine 12∶12 hour light/dark cycle with *ad libitum* access to food and water. The main purpose of using female rats in this study was that one of the common sequelae of SCI is neurogenic bladder. This results in overflow incontinence and loss of voluntary bladder control. Bladder distention and subsequent rupture is more common, and has a higher mortality rate, in males.

### Neural Stem Cells

**Figure 1:**
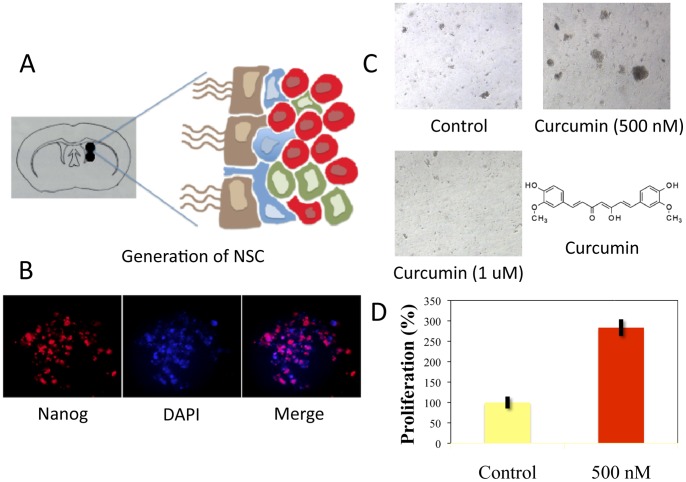
Curcumin enhances stem cell proliferation of NSC derived from SVZ in low doses, however it induces apoptosis at high doses. **A:** Schematic presentation of treatments timeline used in moderate and severe SCI. **B:** Micropunched area of the subventricular zone (SVZ), depicting the presence of ependymal cells (brown), astrocytes (blue), transit amplifying cells (green) and neuroblasts (red). **C:** Immunofluorescence analysis demonstrates the presence of stem cell marker nanog in neurospheres and DAPI staining for DNA. **D, E:** Effect of curcumin on neurosphere formation. At a low concentration of 500 nM, curcumin treatment stimulated proliferation of NSC in 24 hr. At higher concentrations (1 uM), curcumin caused autolysis and neurosphere fragmentation. The chemical structure of curcumin is also displayed.

NSC were extracted from the subventricular zone (SVZ) of female Sprague-Dawley rat brains (Fig. 1A). Brain was removed following craniectomy from a euthanized animal and was placed in dissecting media. Brain was sectioned in multiple coronal sections approximately 3–4 microns thick rostral to the optic chiasm. The SVZ area was microdissected using the micropunch technique described previously [28] and areas micropunched as shown in Fig. 1A. The microdissected specimens were then digested using an enzyme digestion solution for 1–3 hrs at 37°C in a water bath. Following enzymatic digestion, the cells were resuspended in a specialized serum-free stem cell media containing growth factors such as epidermal growth factor, fibroblast growth factor, etc., and plated onto 12-well plates. The cells were incubated at 37°C in a 5% CO_2_ chamber. Neurospheres formed in 5–6 days and the expansion was performed as previously described [29]. Neurospheres were stained for the stem cell marker nanog for authenticity, as shown in Fig. 1C.

### Effect of Curcumin on Stem Cell Proliferation (*in Vitro*)

NSC were grown in stem cell media in the presence or absence of curcumin (500 nM or 1 uM). DMSO treated NSCs were used as controls. After 24 hours, neurosphere proliferation was determined by measuring the diameter of each neurosphere.

### Spinal Cord Injury

Surgery was performed as described previously [14], [30]–[32]. Briefly, aseptic T9–T10 laminectomy under anesthesia (pentobarbital sodium; dose 50–80 mg/kg IP; Hospira, Lake Forest, IL) was performed. Spinal cord contusion was induced using a weight-drop apparatus, where a guided 10 g rod was dropped 12.5 or 25 mm onto the exposed dura mater, representing moderate or severe SCI, respectively. Following contusion, the incision was closed with wound clips. The rats received 0.02% amoxicillin in the drinking water to prevent infection throughout the duration of the experiment. Body temperature of 37°C during anesthesia was maintained by a temperature-controlled heating pad and recorded with a rectal thermometer. During the recovery, urine was expressed manually twice daily to assist in urination until intrinsic function returned to normal.

### Stem Cell Transplant

Rats in the stem cell treatment group underwent repeat surgery one week after injury. Under pentobarbital anesthesia (as described above) and sterile conditions, the original incision was reopened and the dura overlying the site of contusion was exposed. 1×10^6^ neural stem cells in 20 ul of sterile PBS were then microinjected directly into the injured spinal cord. Stem cell injections were performed manually over ten minutes using a 30-guage needle. Injections were performed 1 mm into the spinal cord at the site of contusion injury. The incision was then closed in layers.

### Treatments

Rats were randomized into the 3 (moderate SCI) or 4 (Severe SCI) treatment groups as shown in Table 1. The schemas of treatments/protocols for moderate or severe SCI used in this study are described in Fig. 1A.

**Table 1:** Experimental cohorts: number of animals per cohort.

SCI severity	Control (DMSO)	Curcumin	NSC	NSC+Curcumin
Moderate	6	*	7	6
Severe	12	10	9	13

*Published [14].

#### Moderate SCI

Within 30 minutes following the contusion, rats were given either curcumin (60 mg/ml/kg BW in DMSO), or DMSO (1 ml/kg BW) via percutaneous, intramuscular injection near the site of injury. These injections were continued weekly for six weeks. Stem cell transplantation occurred one week following injury via direct injection into the spinal cord at the site of injury as described above. Rats were given either NSC or combinational treatment (NSC+curcumin) (Fig. 1A). Rats were given curcumin alone following this same protocol in a study previously reported by our laboratory [14].

#### Severe SCI

Within 30 minutes following the contusion, rats were given either curcumin (60 mg/ml/kg BW in DMSO), or DMSO (1 ml/kg BW) via percutaneous-intramuscular injection at the site of injury. Rats were given NSC, curcumin, or combinational treatment (NSC+curcumin). Rats given curcumin monotherapy continued to receive weekly injections at 60 mg/ml/kg BW in DMSO. Rats given stem cell transplant, following the initial dose of DMSO or high dose curcumin (60 mg/ml/kg in DMSO), then received daily IP injections of low dose curcumin (0.5 mmol/ml/kg BW), or an equivalent dose of DMSO alone, for two weeks following stem cell transplant. Stem cell transplant occurred one week following SCI.

### Basso, Beattie and Bresnahan Score

Functional tests were performed before the injury and then weekly after the treatment. Locomotor activity was evaluated using the Basso, Beattie and Bresnahan (BBB) scores for hindlimb rat motor function and was recorded weekly [33]. Testing involved placing the rat on a large open field where a natural aversion to open spaces forced the rat to move towards the edges of the field. Two independent blinded examiners observed hind-limb movements and assessed the rats’ locomotor function [33]. Rats that demonstrated any functional recovery of limb movement within 72 hours of contusion injury were excluded from the study, allowing a more uniform cohort [33]. Rats falling more than two standard deviations above or below the mean were excluded from final statistical review. Data used for analysis included both left and right limb movements, which were recorded separately, but averaged in the final analysis. Body weight was also recorded weekly.

### Soleus Muscle Dissection

After 6 weeks, rats were sacrificed under pentobarbital anesthesia (100 mg/kg IP), and were then perfused with formalin prior to dissection. Perfusion fixation occurred in the following manner: a 5 cm incision rostral to caudal down the sternum was made followed by a 3 cm incision medial to lateral in order to cut the diaphragm. Afterwards, a needle attached to a vacuum-pump system was inserted through the apex of the left ventricle into the ascending aorta whereby formalin fixative was perfused into the rodent. Following fixation, a 1 cm incision was made in both posterior hindlimbs to retrieve soleus muscles. First, the Achilles’ tendon was dissected out, and then the gastrocnemius muscle was elevated to expose the soleus muscle. This was identified, removed *en bloc*, and the weight of each soleus muscle was separately recorded, and averaged for final analysis.

### Histopathological Analysis

Spinal cords at the region of injury were fixed in formalin and placed in paraffin blocks. Six µm axial sections were cut and placed onto the slides. The sections were then stained with Lox Blue staining. Spinal cord atrophy was evaluated by determining the epicenter using an Axiovert Zeiss microscope (Carl Zeiss Microscopy, Thornwood, New York) with the AxioVision software. The spinal cord photographs were then measured utilizing ImageJ software to determine the spared tissue area as well as total spinal cord area. The peak contusion site (epicenter) was recorded as the area with the least amount of spared spinal cord tissue. Five different measurements were taken for each rat, which included one for the epicenter and two from either side of the epicenter (cranial and caudal). Total spinal cord values were also recorded for each section, and percentage of spared area in relation to total area was used for final analysis. The presented values are an average for each rat ± SEM (standard error of the mean).

### Statistical Analysis

Data are presented as a mean ±SEM. BBB scores and body weight were analyzed by two-way analysis of variance (2-ANOVA) between groups over time followed by two-tailed t-test. Soleus muscle was analyzed using the two-tailed t-test. A correlation between the BBB scores and body weight and between BBB score and soleus muscle weight was established using the Pearson method. Statistical significance was determined at p≤0.05. For histopathological analysis, the statistical analysis was done using the two-tail T-test between DMSO treated versus other treatments individually.

## Results

### The Effect of Curcumin on NSC Proliferation

The effect of curcumin on NSC proliferation was examined in a cell culture model. NSC, as represented by neurospheres and staining positive with nanog, were grown in stem cell media in the presence or absence of curcumin. At a low concentration, curcumin (500 nM) caused a significant increase in neurosphere proliferation by over 180% in comparison to control. However, curcumin at higher doses (1 uM) caused widespread apoptosis and neurosphere fragmentation (Fig. 1 D, E).

### BBB Score after Moderate SCI

**Figure 2:**
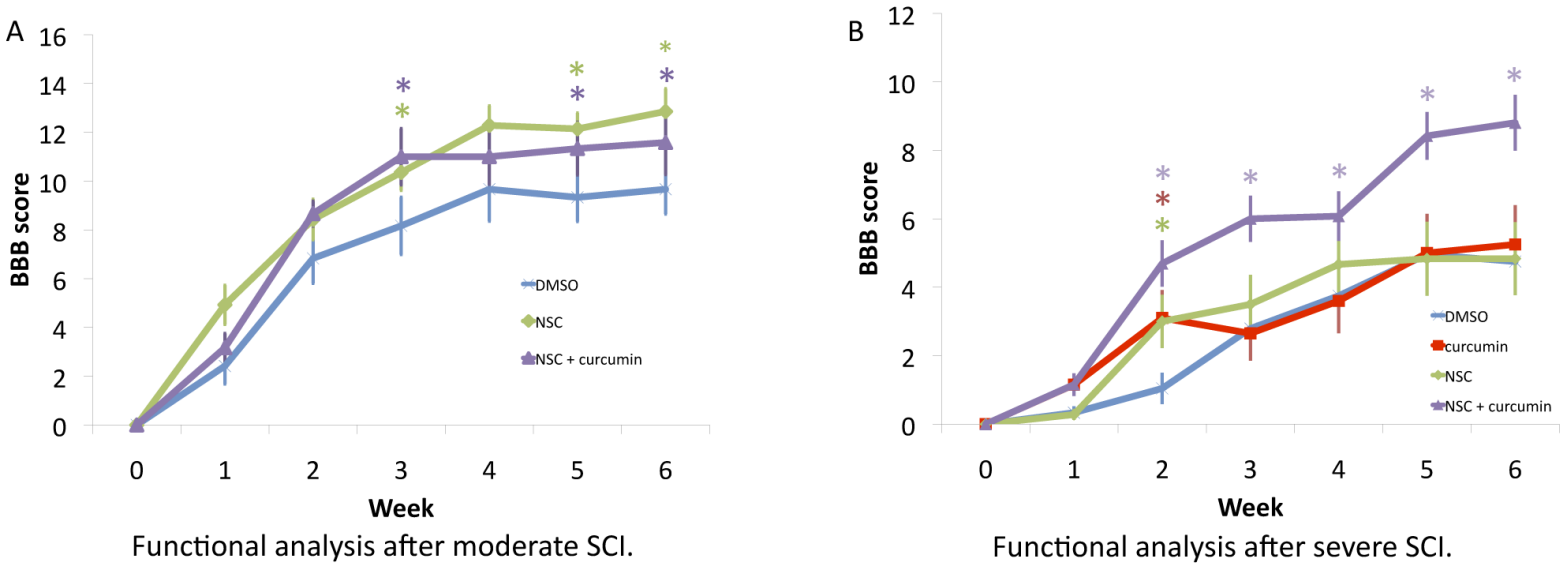
Stem cell therapy in conjunction with low-dose curcumin had a synergistic effect in recovery from severe SCI, as demonstrated by improved BBB score. **A:** Functional analysis (BBB) after moderate SCI showed that stem cell therapy with a high dose of curcumin rendered BBB scores, which were statistically significant as compared to control rats (DMSO), starting from week 3. **B:** In severe SCI, rats undergoing combined NSC transplantation and curcumin showed consistent improvement in BBB score as compared to DMSO treated controls. Statistical analysis of combined NSC+curcumin treatment also demonstrated significant improvement in comparison to NSC or curcumin treatment alone.

Animals undergoing NSC transplant, or with combined treatment using NSC+curcumin, were tested after moderate SCI. Rats treated with NSC transplant demonstrated a significant improvement starting at week three. A significant difference in BBB score between cohorts was seen with the scores of 12.14±0.65 and 9.33±0.99 (p = 0.028) at week five and 12.86±0.93 and 9.67±1.02(p = 0.029) at week six, for NSC treated and control rats, respectively. Combinational treatment with high dose curcumin and NSC transplant significantly improved the BBB score as compared to control. However, between combinational treatment and NSC alone, there was no noticeable difference in improvement based on BBB score (Fig. 2A). Curcumin monotherapy showed significant improvement in recovery from SCI in comparison to control, as we have previously reported [14].

### BBB Score after Severe SCI

Curcumin and NSC treatment demonstrated similar results, in that they both initially demonstrated significant improvement at week two, but this significance was lost through the remainder of the experiment in comparison to controls (Fig. 2B). Rats undergoing combined NSC transplantation and curcumin showed consistent significant improvement in BBB score as compared to controls. This improvement began at week one, where BBB scores were 1.15±0.33 vs. 0.33±0.19 for combinational treatment and control, respectively (p = 0.037), and persisted through week six. While control rats continued to show some improvement over time, the combined NSC/curcumin cohort displayed more rapid and elevated BBB scores in comparison to controls. In fact, by week six, animals continued this significant difference with BBB scores of 8.81±0.82 and 4.75±0.78, for combinational treatment and control, respectively (p = 0.0001). Statistical analysis of combined NSC/curcumin treatment also demonstrated significant improvement in comparison to NSC or curcumin treatment alone.

### Body Weight Gain after Moderate SCI

**Figure 3:**
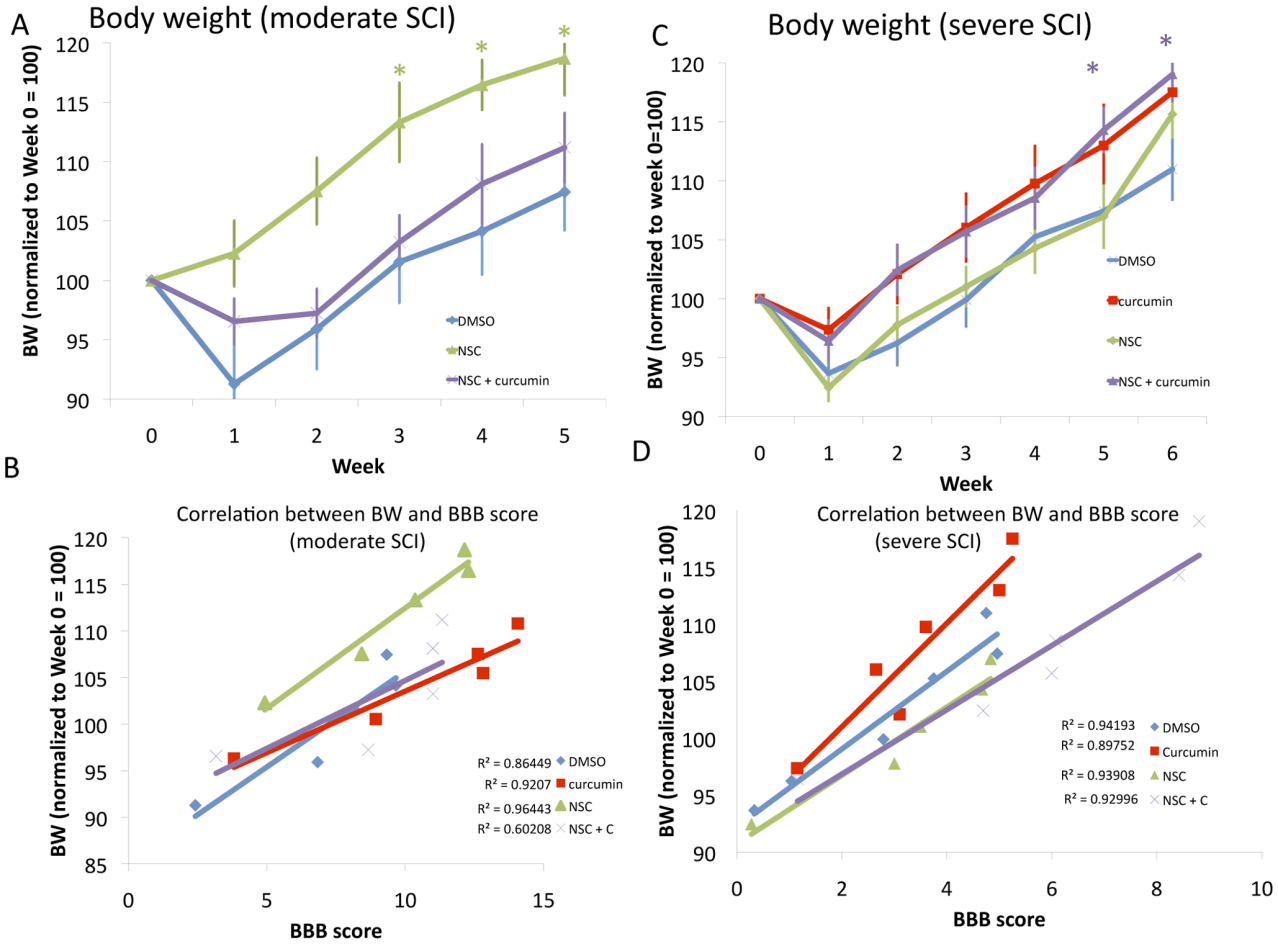
Stem cell therapy in conjunction with curcumin synergistically improves recovery from moderate or severe SCI, as demonstrated by improved body weight gain, which correlated with BBB score. **A:** Stem cell transplanted animals showed improved body weight gain in comparison to combinational treatment or DMSO treated controls. **B:** A positive correlation exists between body weight gain and improved BBB score in moderate SCI. R^2^ values are listed next to their respective cohort. **C:** Combination of NSC therapy with curcumin treatment showed significant improvement in body weight gain in comparison to DMSO control. Curcumin treated rats showed a trend toward significance in BW gain. **D:** Correlation of BBB scores with body weight after severe SCI. R^2^ values are listed next to their respective cohort.

Weekly body weight is presented as a percent change from initial body weight recorded immediately prior to SCI. Many animals initially lost weight after SCI, but then began to increase in weight in a manner that often correlated with recovery. Body weight gain became statistically significant in NSC treated rats at week three and remained significant through week 5 (Fig. 3A). Combined treatment of NSC and curcumin in high doses after moderate SCI showed no statistically significant benefit in body weight gain in comparison to control. Correlation analysis once weight gain resumed (starting week two) demonstrated a linear relationship between BBB score and body weight after moderate SCI (Fig. 3B), suggesting a strong correlation between body weight gain and BBB score.

### Body Weight Gain after Severe SCI

**Figure 4:**
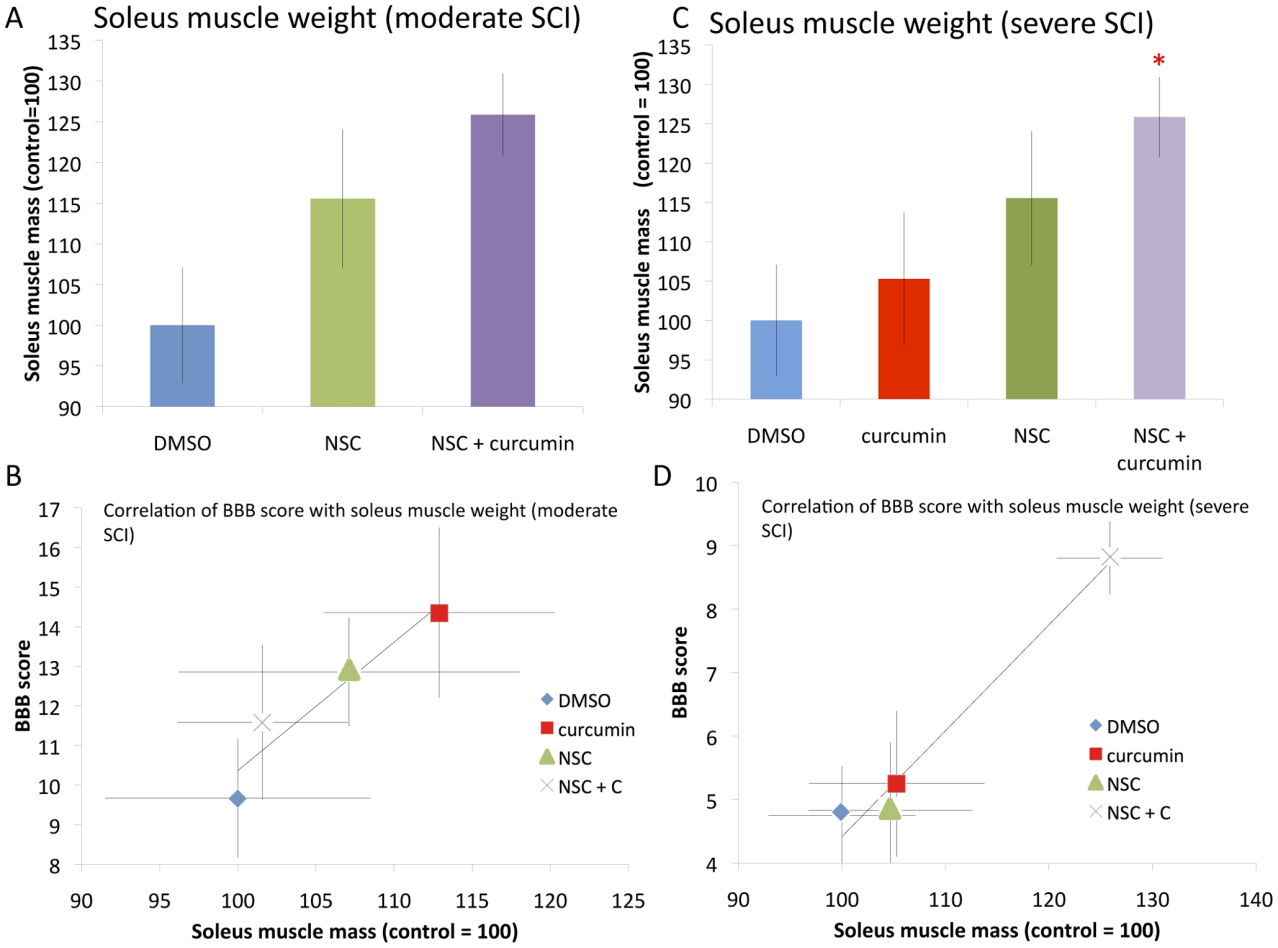
Soleus muscle weight as an indicator of recovery after moderate and severe SCI, and correlated with BBB score. **A:** Rats with greater recovery of hindlimb motor function have better soleus muscle weight at necropsy. While stem cell transplanted animals and combinational treatments had improved soleus muscle weight in comparison to DMSO controls, this did not reach statistical significance. **B:** Correlation of BBB score with soleus muscle weight after moderate SCI demonstrates strong correlation (0.97). Values presented are mean ± SEM. *p<0.05. **C:** Soleus muscle weight after severe SCI showed that all cohorts had larger soleus muscle mass compared to DMSO treated controls, however curcumin or stem cell monotherapy showed only a trend towards significance. NSC plus curcumin treatment improved soleus muscle weight significantly in comparison to DMSO controls. **D:** Correlation of BBB score with soleus muscle weight after severe SCI demonstrated a strong correlation (r^2^ = 0.91). Values presented are mean ± SEM. *p<0.05.

Weekly body weight changes are presented in Figure 3C, where the initial values are set equal to 100, as recorded at the time of contusion. All rats showed a decrease in body weight at week one, as expected, immediately following injury. Combinational treatment showed significance in comparison to control by week five (p = 0.029), curcumin alone showed a trend toward significance (p = 0.20), and NSC treatment showed no significance (p = 0.55). Correlation analysis demonstrated a linear relationship between body weight gain after week one in comparison to BBB score (Fig. 4D).

### Soleus Muscle Mass after Moderate SCI

Soleus muscle weight (mg) was taken at the conclusion of the experiment. Values are reported as a percentage of the control = 100 (see Fig. 4). Rats treated after moderate SCI were not significantly different across cohorts (Fig. 4A). However, NSC treated rats showed higher soleus muscle weight in comparison to control or combined treatment. Specifically, rats had a soleus muscle weight of 100±6.01, 107.12±7.95, and 101.58±13.52 for control, NSC, and combined groups, respectively (p = 0.48 and p = 0.92 for NSC and combinational groups, respectively). Soleus muscle weight correlated with BBB score, implying that higher soleus muscle weight was associated with higher BBB score (Fig. 4B).

### Soleus Muscle Mass after Severe SCI

After severe SCI, control animals had a soleus muscle weight of 100±7.09. Soleus muscle weight was higher in all groups in comparison to control, but only reached significance in the combined treatment cohort. Curcumin had a soleus muscle weight of 105.3±9.46. NSC transplant animals had a soleus weight of 115.54±8.51. Combinational therapy produced a soleus weight of 125.86±5.09. Importantly, soleus muscle weight was significantly better in combined treatment (NSC+curcumin) in comparison to control or in comparison to curcumin or NSC cohorts (p = 0.005, 0.03, and 0.046, respectively) (Fig. 4C). Correlation analysis demonstrated a linear relationship between BBB score and soleus weight (Fig. 4D), demonstrating that the groups showing the most improved BBB score had the greatest soleus muscle mass.

### Histopathological Analysis (Severe SCI)

**Figure 5:**
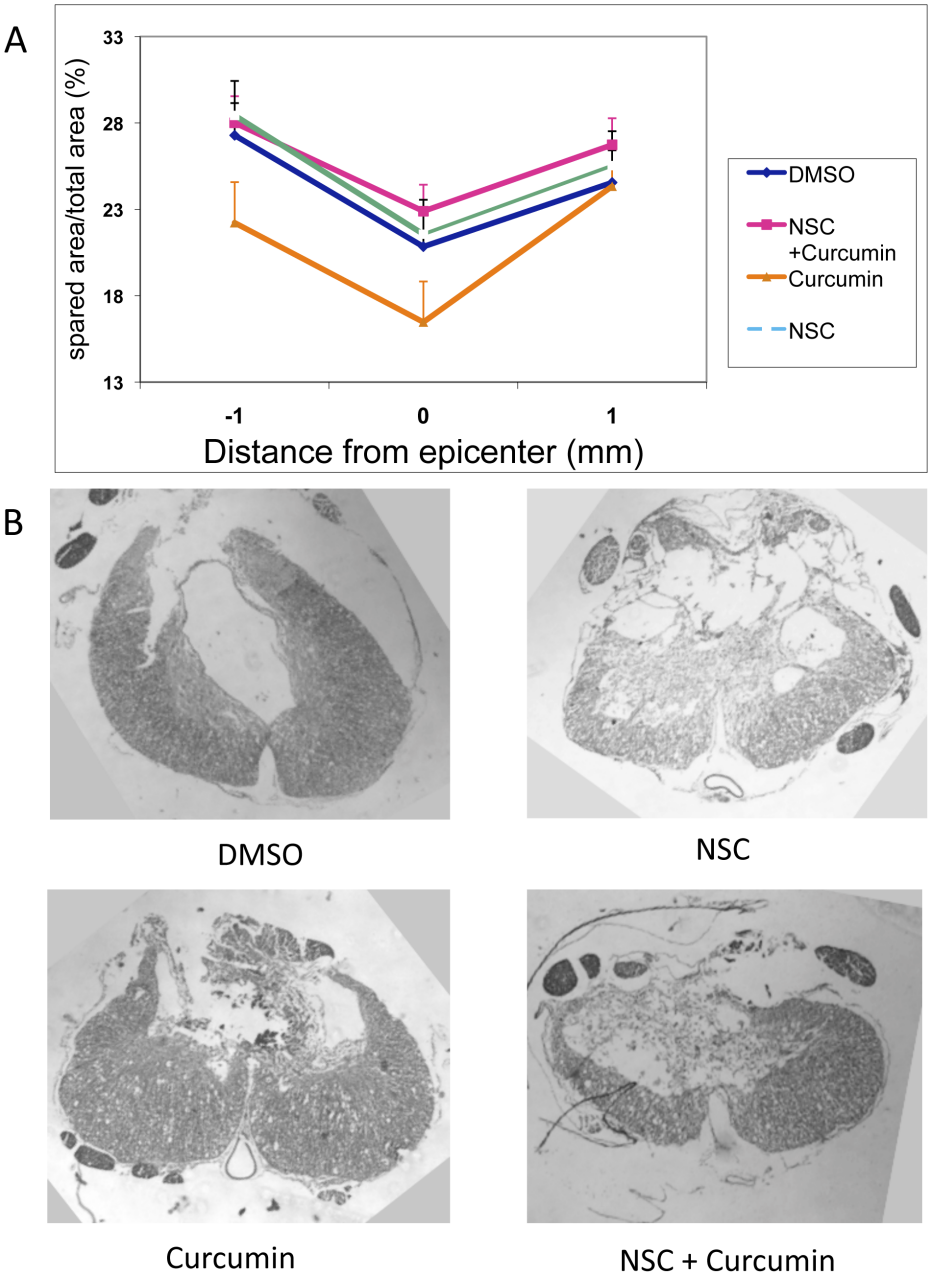
Histopathological examination after severe SCI. **A:** NSC plus curcumin and NSC all displayed enhanced spared area relative to the total area suggesting an improvement in recovery. DMSO treated rats also demonstrated increased spared area of the spinal cord, but somewhat less than the NSC plus curcumin, or NSC treatments. Curcumin-treated rats demonstrated less spared area than the other three treatments. **B:** Photomicrographs of representative samples of spinal cord sections across the area of contusion stained with Lox blue.

Spared tissue in relation to the total area of NSC+Curcumin-, Curcumin-, and NSC-treated rats were presented along with control rats treated with DMSO (Fig. 5A). The rats treated with NSC+Curcumin and NSC monotherapy-treated rats showed better recovery as shown by the spared as compared to the total area.

The statistical analysis using the One Way ANOVA and post test analysis demonstrated no significant difference between the groups (F(3,16) = 1.19; NS), however, it should be noted that curcumin treated rats had the least spared area of the total area as shown in Fig. 5B.

## Discussion

The data presented here demonstrate that NSC transplantation with curcumin synergistically enhanced recovery from severe SCI in a rat model. Recovery from SCI was demonstrated by improvement in parameters including BBB score, body weight, and soleus muscle weight. Stem cell therapies have been shown to be a promising way to regenerate neurons and oligodendrocytes lost during SCI, and have demonstrated efficacy in multiple animal studies [21]–[23], [26], [27]. This is an emerging field in spinal cord injury research, with several human studies currently being conducted worldwide [34]. Our findings differentiate the beneficial effects of NSC transplant by severity of injury. Although NSC treatment alone was sufficient to induce statistically significant recovery after moderate SCI, it could not induce significant recovery after severe injury except in combination with curcumin.

The rationale behind combinational therapy using stem cells and curcumin originates from the idea that curcumin, an anti-inflammatory compound, could reduce gliosis, while stem cells aid in reinervation by increasing the availability of neurotrophic factors at the site of injury. In addition, stem cells may also contribute oligodendrocytes, glia, and neurons to aid in recovery [21], [22], [23], [26], [27]. We have previously demonstrated the benefit of curcumin in moderate SCI where curcumin-treated rats showed marked improvement in functional recovery as early as the first week [14]. Consistently, this study demonstrated that after severe SCI there also was an initial benefit using curcumin. However, this effect was unsustained beyond week two (Fig. 3). Interestingly, combined stem cell treatment with low dose curcumin (0.5 mM/IP daily) significantly improved functional recovery starting from week 2, resulting in many animals recovering to weight bearing status even after severe SCI (Fig. 2B). It should be noted here that this effect of curcumin with stem cell transplant might not be attributable to its anti-inflammatory effect alone. A recent study demonstrated that low dose curcumin treatment increased the number of BrdU-labeled cells in mouse hippocampus, suggesting an enhanced proliferative effect *in vitro*, as well as *in vivo* [35]. Consistent with these findings, we observed curcumin in low doses induced stem cell proliferation *in vitro* (Fig. 1D). Curcumin, however, in higher doses (1 uM) caused apoptosis of stem cells. This proliferative effect may be one reason there is a synergistic effect of combinational treatment on SCI recovery. The lack of benefit of high dose curcumin in combination with stem cell transplant in the moderate injury cohort may also be explained by these findings, where the NSC+high dose curcumin cohort recovered less well than NSC treatment alone (Fig. 3A). Thus, it is possible that curcumin may not only aid the local cellular microenvironment, but also cause proliferation of transplanted stem cells in low doses.

Body weight and soleus muscle weight were used as surrogate markers of recovery following moderate or severe SCI. There was a decrease in body weight during the first week following SCI, however an increase in body weight began starting the second week in a manner that often correlated with functional recovery [14], [30]–[33]. Combinational treatment with curcumin in high doses also demonstrated similar results after moderate injury, and did not significantly differ from controls. Combinational treatment improved body weight gain in a statistically significant manner following severe SCI. These data all correlate well with BBB score findings. Soleus muscle weight has also been used in previous studies as a surrogate marker of recovery of hindlimb motor function [14], [30]–[33]. Following SCI, muscle atrophy is more pronounced in paralyzed muscles that contain a large proportion of slow fatigue-resistant muscle fibers, such as the soleus muscle, that are largely responsible for maintaining posture and bearing weight [36]. Therefore, soleus muscle atrophies more quickly and recovers more slowly than other hindlimb muscles [37]. It also is anatomically simple to isolate and remove completely for assessing its mass [14], [30]–[33], [46]. Additionally, functional recovery following SCI, frequently measured by the BBB score, has been positively correlated to soleus muscle changes including improvement in muscle mass, MRI T2 changes, electrophysiological testing parameters, and a change in the proportion of slow Type 1 fibers [14], [30]–[33], [36]–[40]. This implies that enhanced locomotor activity leads to an improvement in muscle mass. In our study, soleus muscle mass also demonstrated improvement in stem cell transplanted animals after severe SCI in a statistically significant manner (Fig. 5). Correlation data also demonstrated significant correlation between BBB score and soleus muscle mass or body weight, adding credibility to the findings of significant synergistic improvement after severe SCI (Fig. 4–5).

Histopathology data somewhat correlated with functional outcomes. Combined treatment, with the best functional outcome, also had the largest spared tissue. NSC transplant monotherapy also fared better than control, although functional outcomes were not significantly different. Of note, curcumin monotherapy had the least spared tissue among the severe SCI cohorts, but functionally showed similar outcomes to control and NSC transplant alone. It should be emphasized that multiple previous studies have found no relation between functional outcome and the amount of spared tissue at the site of injury [42]–[44]. In general, these data serve to corroborate findings of BBB score, body weight, and soleus muscle weight, supporting the conclusion that improved functional outcomes have a physiologic correlation.

Various types of stem cells have been used for transplantation after SCI. These cell types most commonly include embryonic and adult NSC, fate-restricted neural-glial precursor cells, olfactory ensheathing glial cells, Schwann cells, mesenchymal stem cells, and bone-marrow stromal cells [20]. Adult NSC have been previously characterized in mammalian brain, and are abundant in the SVZ and dentate gyrus [27], [45]–[48]. Endogenous NSC have even been found to exist in the spinal cord, although oligodendrocyte differentiation of these cells has not proven sufficient to promote remyelination after SCI [21]–[23], [47]. This remains true even following infusion of exogenous growth factors [49]–[50]. More recently, however, studies have provided evidence of functional motor recovery after transplantation of ependymal stem progenitor cells that were derived from adult rat spinal cord with a traumatic lesion [51]. In this study, cells were propagated and differentiated *in vitro* into oligodendrocyte precursor cells (OPCs) and engrafted after cell transplantation. Endogenous or transplanted NSCs can differentiate into oligodendrocytes and astrocytes, contributing to remyelination of axons and aiding in recovery [52]. While recruitment of these endogenous NSCs can occur as a part of spontaneous recovery, transplantation of NSCs provides an improved strategy for a faster and better recovery after SCI.

Adult neural stem cells are most commonly derived from the SVZ of the brain and have been found to differentiate to neurons, glia, and oligodendrocytes, although neuronal marker expression is generally rare [20]. This may be due to the local micro-environment at the site of injury, that may affect the differentiation of transplanted NSC. These adult NSC have been found to generate compact myelin in the injured spinal cord, although this has been somewhat inconsistently reported [20]–[23]. The choice of using adult NSC is due to their decreased risk of tumorigenesis [20]–[22], their ability to differentiate along a neural lineage with great consistency [18], [20]–[23], [26], [53], [54], and their repeated benefit to recovery using a thoracic contusion model with behavioral recovery evaluated with the open field BBB score [21]–[23], [55]–[57]. All but one of these studies delivered stem cells directly to the injured spinal cord, similar to what was performed in this study [20], [56].

Secondary damage to the spinal cord following SCI is induced primarily by inflammation and ischemia/reperfusion injury. White matter is especially susceptible to ischemic damage, especially by lipid peroxidation [58], [59]. Oligodendrocytes are also vulnerable to superoxide produced during ischemia because these cells only contain low levels of superoxide antagonists like Mu-superoxide and catalase [58], [60]. Apoptosis also occurs due, in part at least, to the inflammatory cascade, influx of calcium, and the activation of the caspase cascade [58], [61], [62]. A number of agents have been used to reduce ischemia/reperfusion injury to the spinal cord with varying degrees of success including statins, calcium channel blockers, and caspase inhibitors, among others [58], [61], [62]. These studies demonstrate the risk of further SCI secondary to ischemia/reperfusion, and the possible benefits of using agents for their neuroprotective effects.

Curcumin is a potent antioxidant and free radical scavenger, and has been shown to improve ischemia-reperfusion injury in multiple studies [63]–[66]. Curcumin has a potent inhibitory effect on the NF-kB pathway in settings where it is activated [67]–[69]. NF-kB has been demonstrated to be highly upregulated in SCI, and is associated with glial scar formation [70]–[71]. Curcumin may exert its benefit in SCI recovery via inhibition of this pathway [9]–[12], [14]. Absorbtion of curcumin in the GI tract has been debatable, and once absorbed into the blood stream, detectable levels are transient and require very high oral doses to achieve [72]. In order to alleviate the difficulties of poor bioavailability of curcumin, we dissolved curcumin in DMSO as a solvent to increase the rate of transfer across membranes, and administered it by IP injection or locally at the site of injury. Side effects of curcumin are limited, with no side effects noted in our animals [73]. It is noteworthy that despite the previously noted studies demonstrating curcumin’s benefit after SCI, this study extends the findings by demonstrating that this benefit appears to be limited depending on the severity of injury [9]–[12], [14]. For example, in severe injury, curcumin only benefitted recovery following combinational therapy with NSC. Limitations to recovery with existing therapies are not completely surprising, and have been noted in SCI studies in the past with other agents [74]–[76].

Our study demonstrates some of the limitations not only of curcumin, but also stem cell monotherapy, on recovery from SCI. While curcumin’s bioavailability is limited, this may be useful where it is used in combinational therapy [35], [73]–[74]. There is evidence, for example, that only very low concentrations of curcumin following IP injection cross the blood brain barrier [35], [73]–[74]. However, it is precisely this low dose of curcumin that creates the synergistic effect seen in our study. A higher dose of curcumin in combination with NSC had a less significant effect than either therapy alone (Fig. 2A). These results emphasize that the synergistic effect of curcumin with stem cell therapy in severe SCI may occur via dual action of curcumin, i.e. it reduces the inflammation at the local SCI region induced by secondary injury and may also induce proliferation of stem cells required for faster regeneration. With the great interest in stem cell transplant after SCI, it is important to know what adjunctive agents may serve to enhance their effect. This is the first study to demonstrate significant functional recovery from severe SCI in animals treated with curcumin in combination with NSC transplant. Many of these animals regained independent ambulatory function even after severe SCI, a feat not accomplished with either therapy alone. Curcumin and NSC transplant appear to be a safe and effective treatment to improve recovery from SCI. Future studies would include the analysis of discrete biomarkers associated with inflammation and recovery after curcumin treatment.

### Conclusions

The results of this study provide a strong basis for stem cell therapy in conjunction with curcumin in the treatment and management of SCI in humans.
